# Description of two new species and redescription of one species of agnarid terrestrial isopods (Oniscidea, Agnaridae) from western Iran

**DOI:** 10.3897/zookeys.440.7407

**Published:** 2014-09-15

**Authors:** Ghasem M. Kashani

**Affiliations:** 1 Department of Biology, Faculty of Science, University of Zanjan, Zanjan, Iran

**Keywords:** Oniscidea, Agnaridae, new species, Iran

## Abstract

The present study reports on three species of terrestrial isopods from western Iran. The genus *Mongoloniscus* Verhoeff, 1930 is recorded for the first time from Iran, with description of a new species: *M. persicus* **sp. n.** *Protracheoniscus ehsani* **sp. n.** is described and *P. darevskii* Borutzky, 1975 is redescribed based on Iranian specimens. The diagnostic characters of these species are figured and their geographical distribution is presented on a map.

## Introduction

The terrestrial isopods of the family Agnaridae Schmidt, 2003 are distributed from the Mediterranean region to eastern and southern Asia (Schmidt 2003, 2008). The German author postulated an Indian origin for the family. He considered the internal lungs with spiracles located on the lateral margin of all pleopod exopodites as the only autapomorphy of the family.

**Figure 1.**
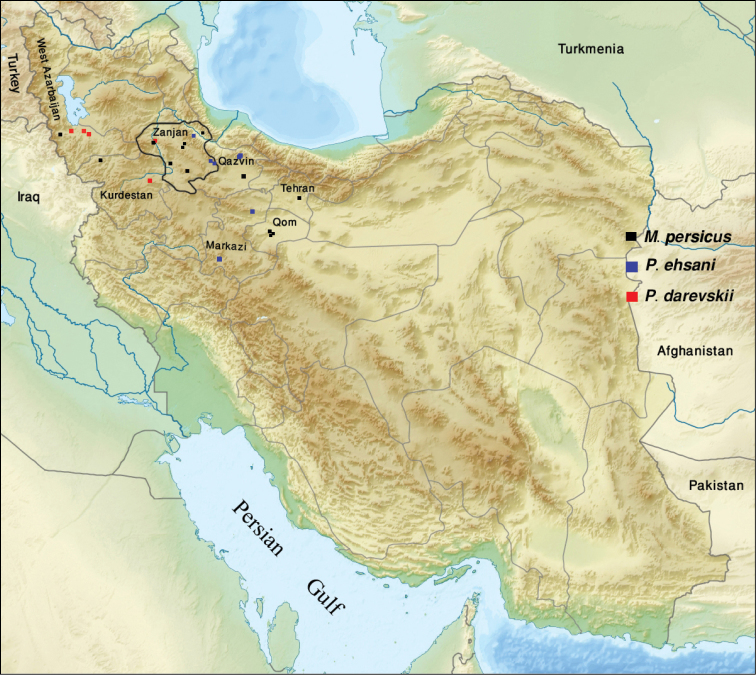
Map of Iran indicating the sampling localities of *Mongoloniscus persicus* sp. n. (in black), *Protracheoniscus ehsani* sp. n. (in blue) and *Protracheoniscus darevskii* (in red).

According to world catalogue of terrestrial isopods (Schmalfuss 2003), Agnaridae include 15 nominal genera. Former studies recorded two genera in Iran: *Hemilepistus* Budde-Lund, 1879 and *Protracheoniscus* Verhoeff, 1917 (Kashani et al. 2010, 2013, Kashani 2014). In this study, three agnarid species are reported from western Iran, of which two are new species. The genus *Mongoloniscus* is found for the first time in Iran. *Protracheoniscus darevskii* Borutzky, 1975 is reported for the first time from Iran. Since the type specimens are lost, a redescription of the species is presented based on Iranian specimens. Moreover, two new species, namely *Mongoloniscus persicus* sp. n. and *Protracheoniscus ehsani* sp. n. are described. Sampling localities for these species are presented on the map (Fig. 1).

## Material and methods

The material examined was collected in many localities from western Iran (Fig. 1). Specimens were collected by hand and preserved in 96% ethanol. The isopods were dissected and body parts were slide-mounted in Euparal. Drawings were made using a drawing tube fitted on a SaIran ZSM-100 dissecting stereomicroscope and on a Nikon Y-IDT compound microscope. Type material of the newly described species is deposited in the Zoological Museum, University of Tehran (ZUTC), Staatliches Museum für Naturkunde, Stuttgart (SMNS), Iranian Research Institute of Plant Protection (IRIPP) and in the author personal collection (PCGMK). All the other specimens are kept in PCGMK.

## Taxonomy

### Order Isopoda Latreille, 1817
Suborder Oniscidea Latreille, 1802
Family Agnaridae Schmidt, 2003

#### Genus Mongoloniscus

Taxon classificationAnimaliaIsopodaAgnaridae

Verhoeff, 1930

##### Diagnosis.

Kwon (1993) discussed in details the characteristics of the genus *Mongoloniscus* and considered it as a good genus. He mentioned the granulated dorsum and triangular median lobe of the head as differentiating characters of the genus from *Protracheoniscus*. According to the eco-morphological classification proposed by Schmalfuss (1984), the members of the genus are clinger type.

#### Mongoloniscus persicus sp. n.

Taxon classificationAnimaliaIsopodaAgnaridae

http://zoobank.org/7AD9DA60-17E6-418B-AEE6-346289578E08

##### Material examined.

Holotype: male, 5.5 mm, **Zanjan**, the University of Zanjan campus, 13 June 2011, leg. G.M. Kashani (ZUTC Iso.1121).

Paratypes: **Zanjan**, same data as holotype, two males and two females (IRIPP Iso-1051); same data as holotype, five males and six females (PCGMK1530); Mellat Park, 36°39.5'N, 48°31.5'E, 19 September 2011, leg. G.M. Kashani, one male (SMNS T308); Mellat Park, 36°39.5'N, 48°31.5'E, 19 September 2011, leg. G.M. Kashani, one female (SMNS T309); Mellat Park, 36°39.5'N, 48°31.5'E, 19 September 2011, leg. G.M. Kashani, one female (PCGMK1534); Mahneshan, 26 March 2012, leg. R. Sayadi, three males and five females (PCGMK1535); Mahneshan, 27 March 2012, leg. R. Sayadi, two males and eight females (PCGMK1536); Mahneshan, 25 April 2012, leg. R. Sayadi, ten males and ten females (PCGMK1540); Mahneshan, 26 April 2012, leg. R. Sayadi, two males and one female (PCGMK1541); Mahneshan, 26 April 2012, leg. R. Sayadi, two males and one female (IRIPP Iso-1046); Mahneshan, 6 July 2011, leg. Z. Rostami, three males and two females (PCGMK1597); Tarom, 4 April 2012, leg. A. Ayoubi, six males and five females (PCGMK1519); Qeydar, Panjeh-Ali Mount, 18 April 2013, two males and four females (PCGMK1609); Taham Dam, 6 Km to Golahrood Village, 28 April 2013, five males and six females (PCGMK1611); 10 Km N Halab, 36°18.7'N, 48°07.0'E, 29 September 2008, leg. G.M. Kashani & E. Entezari, three males and one female (PCGMK1715); **Kurdestan**, Saghez to Saheb, 36°12.0'N, 46°25.6'E, 1 October 2008, leg. G.M. Kashani & E. Entezari, two males and two females (PCGMK1346); **West Azarbaijan**, Piranshahr to Oshnavieh, Soufian Village, 2 October 2008, leg. G.M. Kashani & E. Entezari, four males and one female (PCGMK1361), **Qazvin**, Boin Zahra, 30 June 2008, leg. G.M. Kashani, one male(PCGMK1627); **Tehran**, Pishva, 35°12.4'N, 51°48.4'E, 24 June 2008, leg. G.M. Kashani, two males, four females and two juvenile (PCGMK1434); **Qom**, Langrood Village, 2 April 2011, leg. G.M. Kashani, two males (PCGMK1593); Qanavat, 1 August 2013, leg. G.M. Kashani, seven males and ten females (PCGMK1678); Qom City, 1 August 2013, leg. G.M. Kashani, two males and two females (PCGMK1679).

##### Diagnosis.

Head with well developed lateral and median lobes. Male pereopod VII ischium with concave ventral margin. Male pleopod exopodite I with a deep hollow at apex.

##### Description.

**Figure 2.**
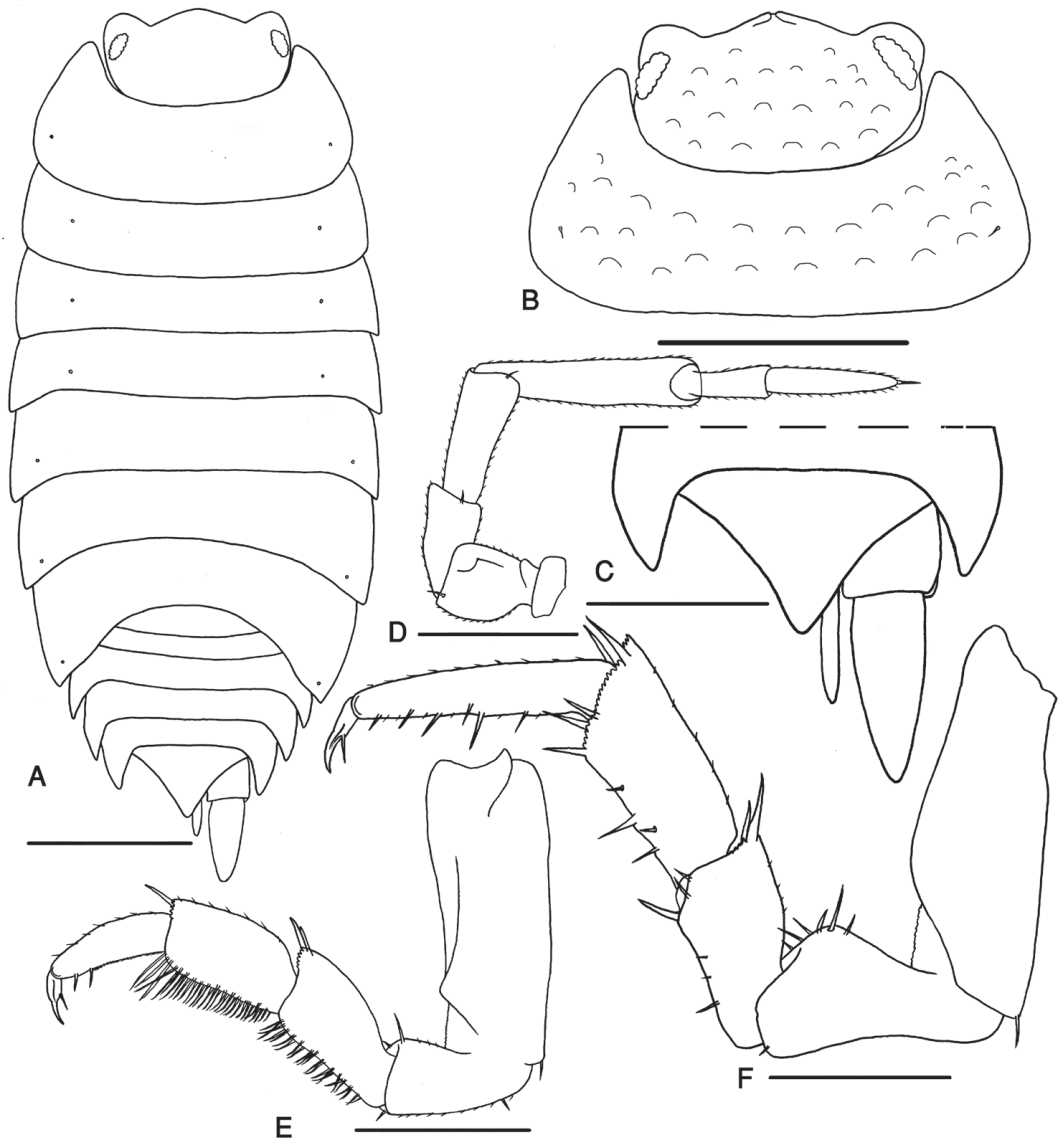
*Mongoloniscus persicus* sp. n., male, paratype. **A** body outline indicating the position of noduli laterales **B** cephalon and first pereonite **C** telson and uropods **D** antenna **E** pereopod 1 **F** pereopod 7. Scale = **A–B** 1 mm; **C–G** 0.5 mm.

Maximum length, male and female, 6 mm. Color pale brown with the usual pale muscles spots. Body outline as in Fig. 2A. Cephalon with well developed lateral and median lobes; frons with an incision in the middle, vertex with faint tubercles (Fig. 2B). Antenna surpassing the posterior margin of pereon-tergite I but not reaching the posterior margin of pereon-tergite II; fifth article of peduncle as long as flagellum, with length:width ratio 4:1; flagellum with two articles, proximal one shorter, flagellar articles ratio 1:1.5 (Fig. 2D).

Pereon covered with faint tubercles. Pereon-tergite I with rounded posterolateral margin. Noduli laterales on pereonites II to IV distinctly more distant from the lateral margins than those on pereonites I and V to VII (Fig. 2A).

**Figure 3.**
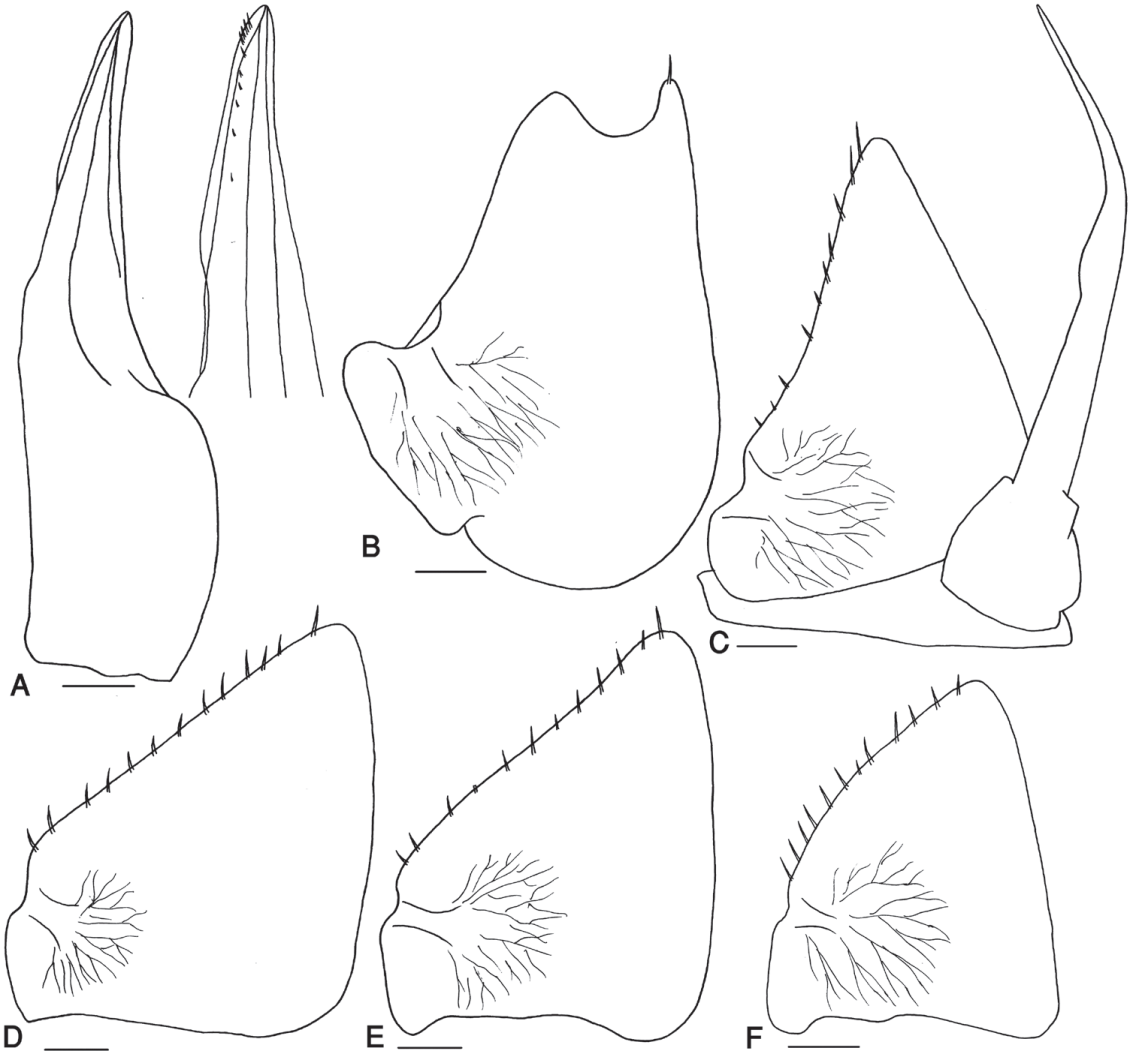
*Mongoloniscus persicus* sp. n., male, paratype. **A** pleopod endopodite 1 **B** pleopod exopodite 1 **C** pleopod 2 **D** pleopod exopodite 3 **E** pleopod exopodite 4 **F** pleopod exopodite 5. Scale = 0.1 mm.

Pleon slightly narrower than pereon (Fig. 2A). Telson triangular with slightly concave sides and rounded apex, surpassing uropod-protopodites but not reaching the middle of uropod-exopodites. Uropod-exopodites short, almost as long as telson (Fig. 2C). Pleopod exopodites I–V with monospiracular covered lungs (Fig. 3B–F).

Male: Pereopods I–III merus and carpus with brushes of trifid setae (Fig. 2E). Pereopod I ischium triangular, carpus with depression on rostral surface equipped with slender scales; propodus narrow and long, proximal part of sternal margin with dense small scales, distal part bearing strong setae; dactylus with one dactylar and one ungual seta (Fig. 2E). Pereopod VII ischium with concave ventral margin, straight in smaller specimens; propodus narrow and long; dactylus with one dactylar and one ungual seta (Fig. 2F). Pleopod exopodite I with long hind lobe bearing a deep hollow and one short seta at apex, outer margin with no setae (Fig. 3B); endopodite I straight with triangular apical part slightly bent outwards and some short setae (Fig. 3A). Pleopod endopodite II longer than exopodite; exopodite triangular with a line of strong setae on outer margin (Fig. 3C). Pleopod exopodites III–V as in Fig. 3D–F.

##### Etymology.

Due to the broad geographical distribution of the species in Iran, the name of the species is after the old name of the country, Persia.

##### Remarks.

Prior to this study, the genus *Mongoloniscus* was only reported from eastern Asia (Kwon 1993; Schmalfuss 2003). *Mongoloniscus persicus* sp. n. is the first species of the genus *Mongoloniscus* to be reported from western Asia. It has a broad geographical distribution in the central and western parts of Iran. Ecologically, this species is well adapted to cultivated areas and exists in huge numbers in some habitats.

##### Distribution.

Iran.

#### Genus Protracheoniscus

Taxon classificationAnimaliaIsopodaAgnaridae

Verhoeff, 1917

##### Diagnosis.

Body length variable, up to 25 mm; tergites always smooth; head with short or developed lateral lobes; antenna variable in size, with flagellum of two articles; pereon epimera I with rounded posterolateral corner; telson triangular with more or less concave sides; male pleopod exopodite I with short to long hind lobe, endopodite I straight; clinger or runner type according to the eco-morphological classification proposed by Schmalfuss (1984).

#### Protracheoniscus darevskii

Taxon classificationAnimaliaIsopodaAgnaridae

Borutzky, 1975

##### Material examined.

**West Azarbaijan**, 58 Km N Mahabad, 37°07.9'N, 45°26.3'E, 4 October 2008, leg. G.M. Kashani & Ehsan Entezari, eleven males, eight females and one juvenile (PCGMK1374); Urumiah to Miandoab, 36°54.7'N, 45°44.9'E, 4 October 2008, leg. G.M. Kashani & E. Entezari, one male and one female (PCGMK1377); 5 Km S Miandoab, 36°56.9'N, 46°09.9'E, 4 October 2008, leg. G.M. Kashani & E. Entezari, one male (PCGMK1381); **Zanjan**, Mahneshan, 26 April 2012, leg. R. Sayadi, one male (PCGMK1546); Mahneshan, 6 July 2011, leg. Z. Rostami, one male and one female (PCGMK1599); **Kurdestan**, 10 Km N Bijar, 30 August 2013, leg. E. Jazimagh, one male and one female (PCGMK1610); 10 Km N Bijar, 25 June 2013, leg. E. Jazimagh, ten males and fifteen female (PCGMK1677); 10 Km N Bijar, 30 August 2013, leg. E. Jazimagh, one male and one female (IRIPP Iso-1047).

##### Diagnosis.

Head with developed rounded lateral and median lobes. Male pereopod VII ischium with straight or concave ventral margin. Male pleopod exopodite I with a deep hollow or obliquely truncate apex. Endopodite I with an apical lobe equipped with small setae.

##### Redescription.

**Figure 4.**
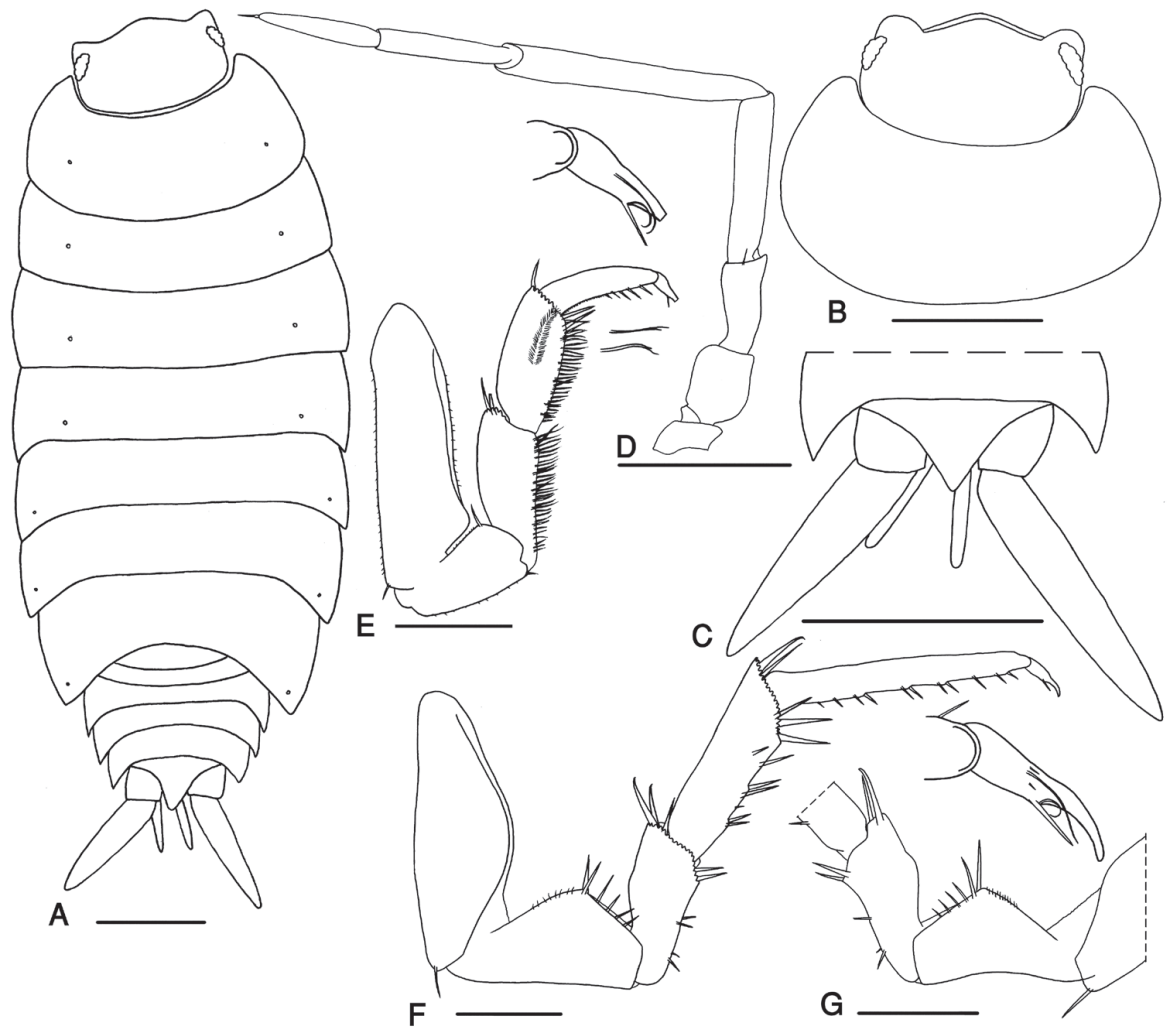
*Protracheoniscus darevskii* Borutzky, 1975, male. **A** body outline indicating the position of noduli laterales **B** cephalon and first pereonite **C** telson and uropods **D** antenna **E** pereopod 1 **F** pereopod 7 **G** pereopod 7 ischium. Scale = **A–C** 1 mm; **D–G** 0.5 mm.

Maximum length, male and female, 10 mm. Color dark brown with the usual pale muscles spots. Body outline as in Fig. 4A. Cephalon with rounded lateral lobes not protruding compared with broadly rounded frons (Fig. 4B). Antenna long, surpassing the posterior margin of pereon-tergite III; fifth article of peduncle as long as flagellum, with length:width ratio 7:1; flagellum with two articles, proximal article as long as the distal one (Fig. 4D).

##### Pereon smooth.

Pereon-tergite I with rounded posterolateral margin. Noduli laterales on pereonites I to IV distinctly more distant from the lateral margins than those on pereonites V to VII (Fig. 4A).

**Figure 5.**
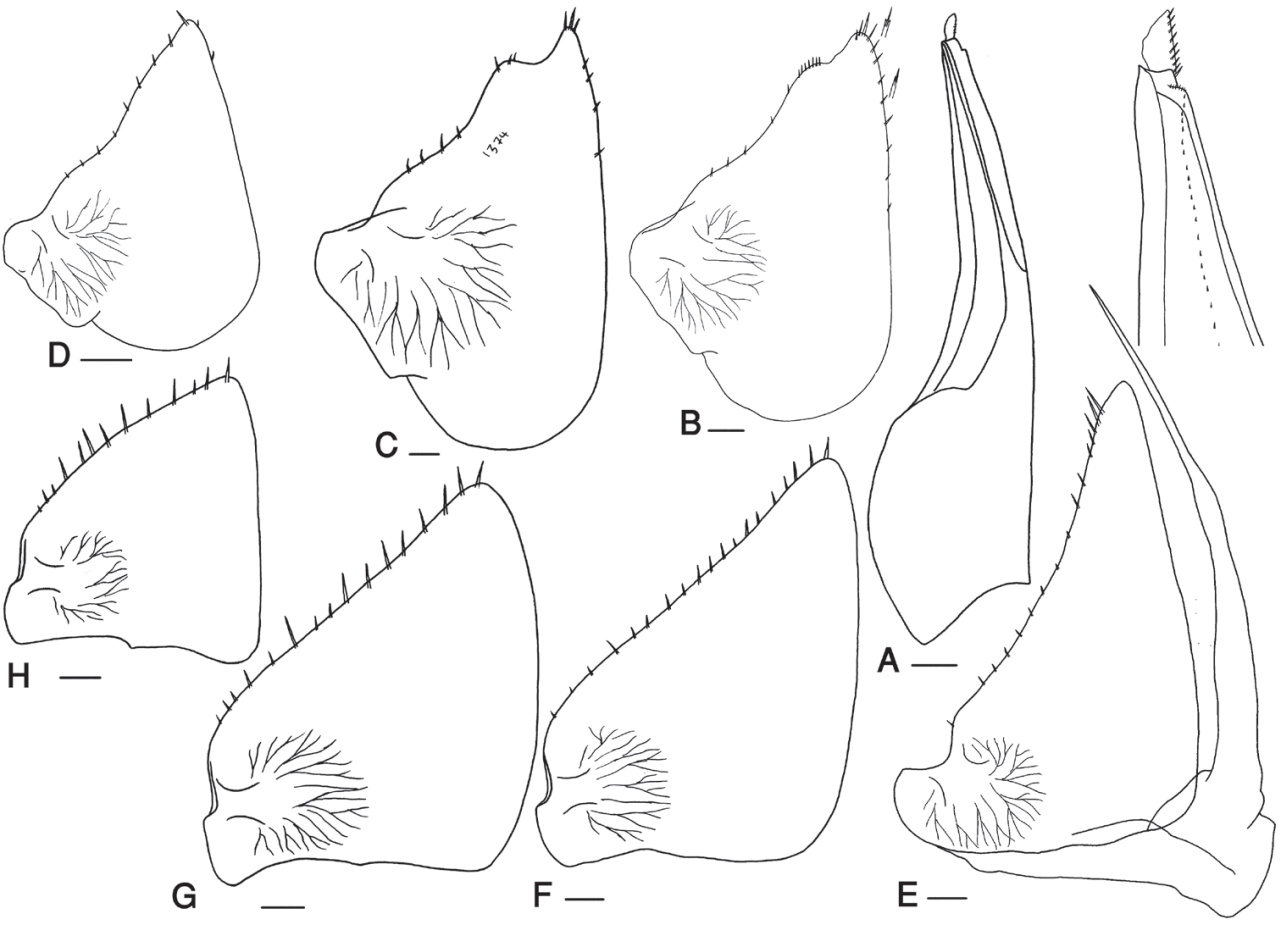
*Protracheoniscus darevskii* Borutzky, 1975, male. **A** pleopod endopodite 1 **B–D** pleopod exopodite 1 **E** pleopod 2 **F** pleopod exopodite 3 **G** pleopod exopodite 4 **H** pleopod exopodite 5. Scale = 0.1 mm.

Pleon narrower than pereon (Fig. 4A). Telson triangular with slightly concave sides and acute distal part (Fig. 4C). Uropod exopodites long, almost 2.5 times as long as telson (Fig. 4C). Pleopod exopodites I–V with monospiracular covered lungs (Fig. 5B–H).

Male: Pereopods I–III merus and carpus with brushes of setae (Fig. 4E). Pereopod I ischium triangular, carpus with depression on rostral surface equipped with slender scales; propodus narrow and long, proximal part of sternal margin with dense small scales, distal part bearing strong setae; dactylus with one dactylar and one ungual seta (Fig. 4E). Pereopod VII ischium with straight or concave ventral margin, merus with a short crest on dorsal margin, propodus narrow and long, dactylus with one dactylar and one ungual seta (Fig. 4F, G). Pleopod exopodite I with long hind lobe bearing a deep hollow at apex, in smaller specimens with an obliquely truncate apex; outer margin with several spine setae (Fig. 5B–D); endopodite I straight with an apical lobe equipped with small setae (Fig. 5A). Pleopod endopodite II longer than exopodite; exopodite triangular with a line of strong setae on outer margin (Fig. 5E). Pleopod exopodites III–V as in Fig. 5F–H.

##### Remarks.

During the examination of type material of terrestrial isopods deposited in Zoological Museum of Moscow State University (ZMMU), it was revealed that the type material of *Protracheoniscus darevskii* is possibly lost. Borutzky (1975) described the species from Armenia and figured its diagnostic characters. *Protracheoniscus darevskii* is here redescribed on the new material from western Iran (Fig. 1).

This species is characterized by the male pleopod exopodite I possessing a deep hollow at apical part of distal margin and endopodite I with an apical lobe bearing small setae.

##### Distribution.

Southern Armenia: Megri District; western Iran.

#### Protracheoniscus ehsani sp. n.

Taxon classificationAnimaliaIsopodaAgnaridae

http://zoobank.org/2860DC91-D00D-41BE-B230-A51943AE76D0

##### Material examined.

Holotype: male, 8 mm, **Markazi**, Saveh to Boin-Zahra, Vardeh, 35°15.3'N, 50°16.5'E, 18 July 2013, leg. G.M. Kashani & B. Eshaghi (ZUTC Iso.1122).

Paratypes: **Markazi**, same data as holotype, one male and one female (IRIPP Iso.1049); same data as holotype, five males and seven females, some with marsupium (PCGMK 1652); Shazand, 9 October 2004, leg. G.M. Kashani, one male (PCGMK 1109); **Qazvin**, 20 Km N Qazvin, 36°20.7'N, 50°10.7'E, 19 July 2013, leg. G.M. Kashani & B. Eshaghi, one male (SMNS T310); 20 Km N Qazvin, 36°20.7'N, 50°10.7'E, 19 July 2013, leg. G.M. Kashani & B. Eshaghi, one female with marsupium (SMNS T311); 20 Km N Qazvin, 36°20.7'N, 50°10.7'E, 19 July 2013, leg. G.M. Kashani & B. Eshaghi, two males and seven females (PCGMK 1675); Qazvin to Razmian, Barajin village, 19 July 2013, leg. G.M. Kashani & B. Eshaghi, two females (IRIPP Iso.1048); Qazvin to Razmian, Barajin village, 19 July 2013, leg. G.M. Kashani & B. Eshaghi, eight females, two males and seven juveniles (PCGMK 1669); **Zanjan**, 25 km to Chavarzaq from Zanjan, 28 April 2013, leg. G.M. Kashani, one male (PCGMK 1614); 25 km to Chavarzaq from Zanjan, 6 May 2013, leg. G.M. Kashani, one male and five females (PCGMK 1615); Abhar, 36°09.4'N, 49°15.4'E, 12 September 2013, leg. G.M. Kashani & B. Eshaghi, four males and four females (PCGMK 1697); Abhar to Darasajin, 36°03.6'N, 49°13.2'E, 12 September 2013, leg. G.M. Kashani & B. Eshaghi, three males and one female (IRIPP Iso.1050); Abhar to Darasajin, 36°03.6'N, 49°13.2'E, 12 September 2013, leg. G.M. Kashani & B. Eshaghi, five males and four females (PCGMK 1699).

##### Diagnosis.

Head with developed rounded median lobe much more protruding than lateral ones. Male pereopod VII carpus with a triangle ridge on dorsal margin. Male pleopod endopodite I with two rows of long setae at apex.

##### Description.

**Figure 6.**
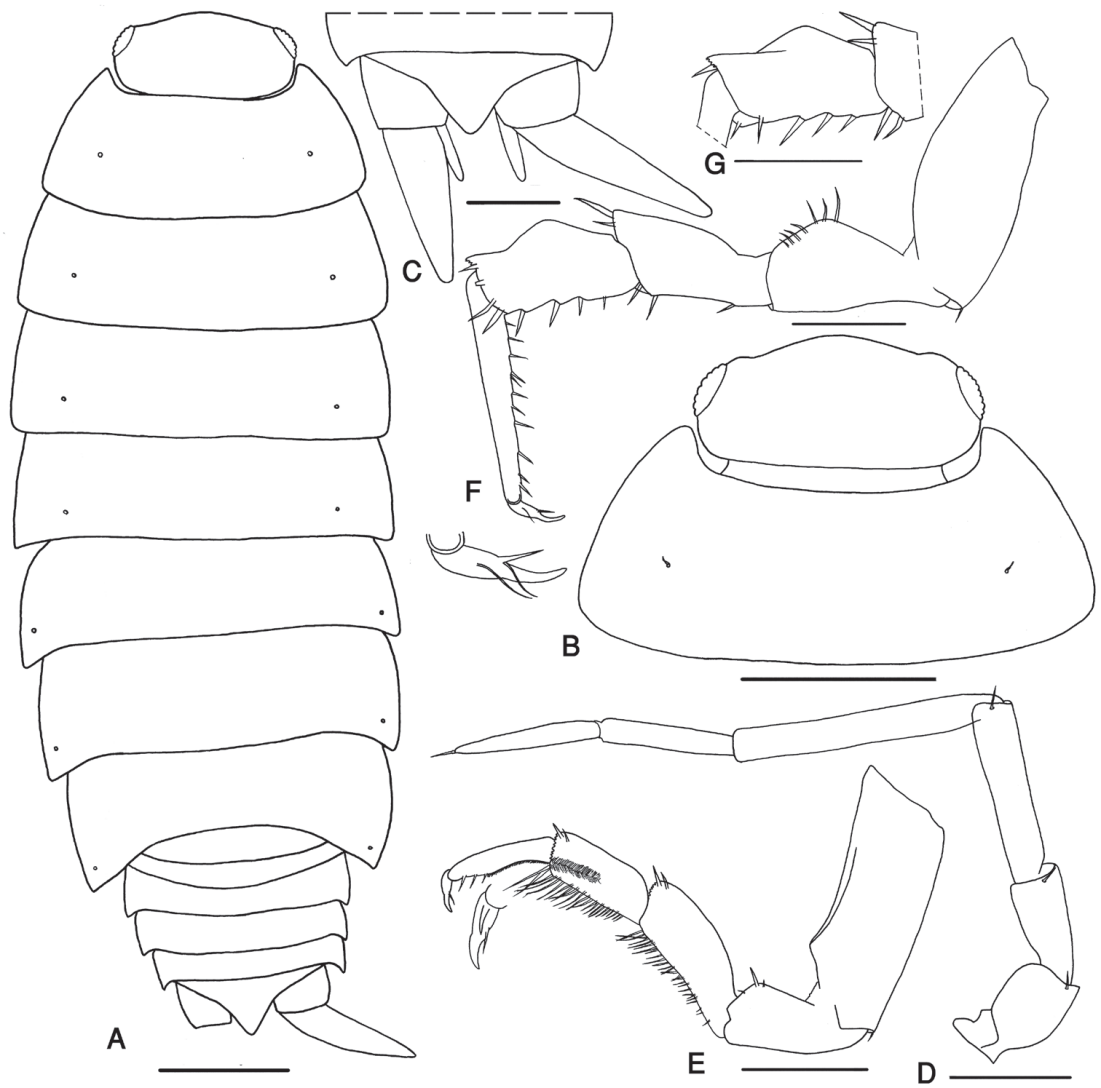
*Protracheoniscus ehsani* sp. n., male, paratype. **A** body outline indicating the position of noduli laterales **B** cephalon and first pereonite **C** telson and uropods **D** antenna **E** pereopod 1 **F** pereopod 7 **G** pereopod 7 ischium. Scale = **A–B** 1 mm; **C–G** 0.5 mm.

Maximum length, male 8 mm and female 11 mm. Color dark brown with the usual pale muscles spots. Body outline as in Fig. 6A. Cephalon with very small lateral lobes not protruding compared with broadly rounded frons (Fig. 6B). Antenna long, surpassing the posterior margin of pereon tergite III; fifth article of peduncle as long as flagellum, with length:width ratio 7:1; flagellum with two articles, proximal article as long as the distal one (Fig. 6D).

##### Pereon smooth.

Pereon tergite I with rounded posterolateral margin. Noduli laterales on pereonites I to IV distinctly more distant from the lateral margins than those on pereonites V to VII (Fig. 6A).

**Figure 7.**
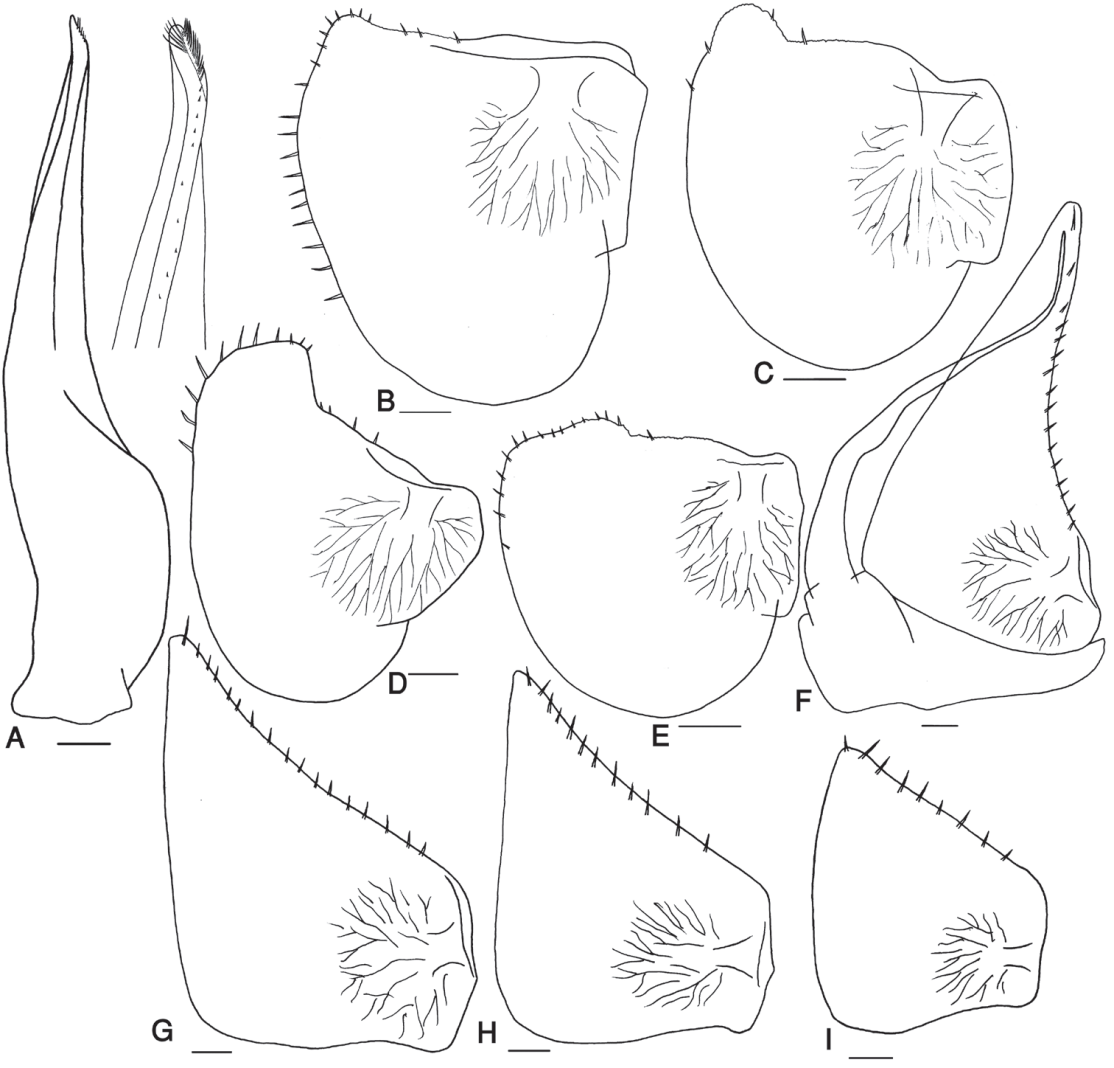
*Protracheoniscus ehsani* sp. n., male, paratype. **A** pleopod endopodite 1 **B–E** pleopod exopodite 1 **F** pleopod 2 **G** pleopod exopodite 3 **H** pleopod exopodite 4 **I** pleopod exopodite 5. Scale = 0.1 mm.

Pleon narrower than pereon (Fig. 6A). Telson triangular in distal part, with rounded apex, slightly surpassing uropod protopodites (Fig. 6C). Uropod exopodites long, almost two times as long as telson (Fig. 6C). Pleopod exopodites I–V with monospiracular covered lungs (Fig. 7B–I).

Male: Pereopods I–III merus and carpus with brushes of setae (Fig. 6E). Pereopod I ischium triangular, carpus with depression on rostral surface equipped with slender scales; propodus narrow and long, proximal part of sternal margin concave with dense small scales, distal part bearing strong setae; dactylus with one dactylar and one ungual seta (Fig. 6E). Pereopod VII ischium with concave ventral margin, carpus with a triangle ridge in dorsal margin, propodus narrow and long, dactylus with one dactylar and one ungual seta (Fig. 6F, G). Pleopod exopodite I hind lobe variable in shape, with rounded short to truncate long distal margin; outer margin equipped with few to several strong setae (Fig. 7B–E); endopodite I straight with apical part slightly bent inwards bearing two rows of long setae (Fig. 7A). Pleopod endopodite II longer than exopodite; exopodite triangular, outer margin convex equipped with a line of strong setae (Fig. 7F). Pleopod exopodites III–V as in Fig. 7G–I.

##### Etymology.

The name of the species is after my late friend, Ehsan Entezari, who unfortunately passed away tragically during a field study.

##### Remarks.

*Protracheoniscus ehsani* sp. n. is characterized by short lateral lobes of head, a triangle ridge on dorsal margin of male pereopod VII carpus, and two rows of long setae at apical part of male pleopod endopodite I. This species is similar to *Protracheoniscus darevskii*, but differs from that in the shape of pleopod endopodite and exopodite I, and in the conspicuous ridge on the dorsal margin of carpus of pereopod VII.

##### Distribution.

Central Iran.

## Supplementary Material

XML Treatment for Mongoloniscus

XML Treatment for Mongoloniscus persicus

XML Treatment for Protracheoniscus

XML Treatment for Protracheoniscus darevskii

XML Treatment for Protracheoniscus ehsani
